# Effects of Oral Hyaluronic Acid Administration in Dogs Following Tibial Tuberosity Advancement Surgery for Cranial Cruciate Ligament Injury

**DOI:** 10.3390/ani11051264

**Published:** 2021-04-27

**Authors:** Claudio Iván Serra Aguado, Juan José Ramos-Plá, Carme Soler, Sergi Segarra, Víctor Moratalla, José Ignacio Redondo

**Affiliations:** 1 Hospital Veterinario UCV, Departamento de Medicina y Cirugía, Facultad de Veterinaria y Ciencias Experimentales, Universidad Católica de Valencia San Vicente Mártir, 46018 Valencia, Spain; mdc.soler@ucv.es; 2 Departamento de Medicina y Cirugía Animal, Facultad de Veterinaria, Universidad Cardenal Herrera-CEU, CEU Universities, 46115 Valencia, Spain; nacho@uchceu.es; 3 R&D Bioiberica S.A.U., 08950 Esplugues de Llobregat, Spain; ssegarra@bioiberica.com; 4 SOT Veterinaria, 46006 Valencia, Spain; sotveterinaria@gmail.com

**Keywords:** hyaluronic acid, synovial fluid, paraoxonase-1, osteoarthritis, canine, cranial cruciate ligament

## Abstract

**Simple Summary:**

Hyaluronic acid injections into the stifle are often used for managing osteoarthritis in dogs. Giving hyaluronic acid orally would be easier, but to date we do not have enough information regarding its effects when administered by this route. For this reason, in this study we evaluated the effects of oral administration of hyaluronic acid in dogs with cranial cruciate ligament rupture after surgical resolution. Dogs were divided into two groups that received either oral hyaluronic acid or a placebo. We measured changes in several biomarkers of osteoarthritis before surgery, and at ten weeks after surgery. Results showed significant improvements in some of these biomarkers, namely synovial fluid levels of hyaluronic acid and paraoxonase-1. These changes indicate that post-op oral administration of hyaluronic acid may be effective for the management of stifle osteoarthritis in the dog.

**Abstract:**

Hyaluronic acid (HA) intraarticular injection is used in the management of osteoarthritis in veterinary medicine. However, HA oral administration is less common given the scarce currently available scientific evidence. This study was aimed at evaluating the effects of oral HA administration on synovial fluid concentrations of several selected biomarkers in dogs with cranial cruciate ligament (CCL) injury operated on using the tibial tuberosity advancement (TTA) technique. Fifty-five dogs were included in this prospective, randomized, double-blind, clinical study; they were randomly assigned to receive either a placebo (group A; n = 25) or HA (group B; n = 30) orally for 10 weeks. Synovial fluid samples were obtained before surgery, and at 10 weeks postoperatively to measure concentrations of HA, haptoglobin, nitric oxide, and paraoxonase-1. After 10 weeks, group HA showed a significant increase in HA concentration (*p* = 0.0016) and a significant decrease in PON-1 concentration (*p* = 0.011) compared to baseline. In conclusion, post-op oral HA administration in canine patients with CCL injury leads to improvements in osteoarthritis biomarkers, namely higher synovial fluid HA concentrations and reduced synovial fluid paraoxonase-1 concentrations. These findings support the bioavailability of orally-administered HA and its usefulness in improving biomarkers of osteoarthritis.

## 1. Introduction

Osteoarthritis (OA) is a complex disease that progressively produces biochemical, structural, mechanical, and functional changes in the synovial joints. The most common abnormalities that may result in the development of secondary OA can be classified as either hereditary or developmental disorders such as osteochondrosis, hip and elbow dysplasia, and patellar luxation, or acquired conditions such as cranial cruciate ligament (CCL) injury [1]. Particularly in dogs, osteoarthritis of the stifle can be considered both the cause and the consequence of cranial cruciate ligament rupture [2].

Treatment of this abnormality is multimodal, addressing the primary root cause and modulating the progression of the secondary disease. Among the various therapeutic options, hyaluronic acid (HA) has shown satisfactory clinical results with a long-lasting effect [3,4,5].

HA is a glycosaminoglycan synthesized by chondrocytes and synoviocytes [6], and it is the main component of synovial fluid and extracellular matrix of cartilage. It generates an appropriate environment for cell migration and proliferation, and confers viscoelastic properties to these tissues [7]. Joints with OA feature a lower concentration and molecular weight of HA [1].

Intra-articular HA injections have been proven effective for the treatment of pain in OA patients [8,9], attenuating lameness, and decreasing augmented movement-induced nerve impulse activity in sensitized joint nociceptor fibers in clinical and experimental OA [10]. In recent years, several studies have assessed the effectiveness of oral HA administration. Absorption and distribution in tissues such as skin, bone, and synovial joints have been confirmed by Balogh et al. (2008) [11] in canine species, and subsequent studies have suggested satisfactory clinical effects in patients with OA [5,12,13]. However, the post-op effects of oral administration of high molecular weight (HMW) HA on synovial concentrations of HA and other biomarkers have not yet been assessed.

Thus, the main aim of this study was to evaluate the effects of oral administration of HMW HA on stifle synovial fluid concentrations of HA, haptoglobin (HAP), nitric oxide (NO), and paraoxonase-1 (PON-1) in dogs with CCL injury operated on using the tibial tuberosity advancement (TTA) technique. Secondarily, another objective of the study was to provide a synovial fluid concentration of several biomarkers (HA, HAP, NO, and PON-1) in those dogs.

## 2. Materials and Methods

### 2.1. Subjects

This was a prospective, randomized (EXCEL^®^, Microsoft, WA, USA), double-blind, clinical study in dogs that had been diagnosed with CCL injury and operated on using the TTA surgical technique.

Healthy dogs between 1 and 10 years of age and weighing between 15 and 45 kg diagnosed with cranial cruciate ligament injury in one of their hind limbs were included in the study. Patients with systemic metabolic disease (such as diabetes and hypothyroidism), immune-mediated diseases, or those that had received immunosuppressive therapy in the two months prior to the study start, and animals with additional stifle injuries (except for meniscal injury) were excluded. Patients that developed complications after CCL surgery and required additional treatment (surgical or medical) were also excluded.

The study was approved by the Ethics Committee for Animal Welfare, in compliance with European guidelines 2010/63/EU. Dog owners were properly informed and gave their written consent.

### 2.2. Dietary Intervention

Dogs were classified into two groups based on the treatment that they received orally: group A (placebo) and group B (HMW-HA). The product used in this study (Mobilee^®^, Bioiberica S.A.U., Barcelona, Spain) is a rooster comb extract especially rich in HMW HA (60–75% HA; 800–1000 kDa), which was formulated in soft gelatin capsules containing 27 mg of HA. Animals weighing up to 26 kg were given one capsule once daily and those weighing more than 26 kg were given two capsules once daily. Neither the clinical nor the laboratory team were aware of group allocation during the course of the study. Treatment administration started 24 h after surgery and continued for 10 weeks.

### 2.3. Experimental Protocol

Patients were evaluated at five different time points (V_0_, V_1_, V_2_, V_3,_ and V_4_), as detailed in Table 1.

The parameters evaluated were as follows:

Functional assessment: by both the owner and the veterinarian. The degree of lameness (EVAP) was evaluated by the owner using a 100-mm visual analogue scale in which 0 was the absence of lameness/pain and 100 the maximum lameness/pain possible. The degree of lameness (VASC) and of pain (VASD) were evaluated by the veterinarian using the same 100-mm visual analogue scale. The veterinarian also evaluated the degree of lameness (EPCOJ) according to the semi-quantitative scale described by Jandi and Schulman (2007) [14] (Lameness scale variable).

**Figure 1:**
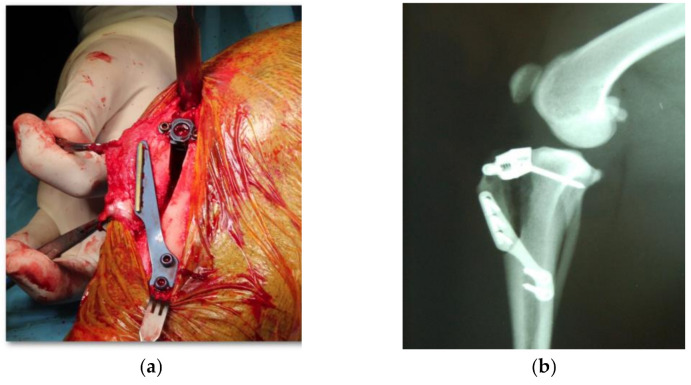
Surgical image of TTA surgical procedure (**a**) and radiologic study image after surgery (**b**).

Surgery: At V_1_, all patients underwent surgery on the CCL injury under general anesthesia, adapting the anesthesia protocol to the patient’s clinical conditions. Arthrotomy was performed on all patients to assess the state of the meniscus, recording the presence or absence of injury as well as the performance or not of a meniscectomy. All patients subsequently underwent TTA surgery, according to the technique described by Montavon et al. (2004) [15] (Figure 1a). A radiological study was performed post-surgery to verify the correct placement of the implants (Figure 1b). Medical treatment for all patients was as follows: 22 mg/kg intraoperative amoxicillin-clavulanic acid (Amoxicilina/Ácido Clavulánico, Normon S.A., Madrid, Spain); 0.1 mg/kg meloxicam (Metacam, Boehringer Ingelheim GmbH, Ingelheim am Reim, Germany) every 24 h for 7 days; and 0.006 mg/kg buprenorphine (Buprex, Quintiles SL, Valencia, Spain) every 8 h for 3 days.

Synovial fluid aspiration: Synovial fluid was aspirated from the operated stifle at V_1_ (baseline) and V_4_ (10 weeks) (Table 1). Aspiration was performed intraoperatively before the arthrotomy at V_1_, and percutaneously under patient sedation at V_4_. A 2-mL syringe (Injekt 2 mL/Luer Solo, BBraun, Melsungen, Germany) and 20G needle (Microdance 3, Beckton Dickinson and Company, Franklin Lakes, NJ, USA) were used.

Synovial fluid analysis: The first 0.5 mL of synovial fluid was placed in an EDTA tube (Aquisel EDTA 3K, Abrera, Barcelona, Spain) and centrifuged at 3500 rpm for 7 min. After centrifugation, the supernatant was removed and placed in an Eppendorf tube and then frozen immediately at −25 °C.

The HA measurement was performed using an ELISA test (TECO^®^ Hyaluronic Acid, TECOmedical, Headquarters, Sissach, Switzerland). The remainder of the synovial fluid was placed in an Eppendorf tube and stored at −25 °C. The concentrations of the remaining biomarkers (HAP, PON-1, and NO, in that order) were measured using these samples. HAP was measured using a commercially-available method, which had been previously validated for dogs (Tridelta PHASE™ Haptoglobin kit, Tridelta Development Ltd., Brey, Ireland), NO was quantified with a Griess reagent according to the modified Miranda protocol, and PON-1 concentration was measured by ELISA (Human PON-1 ELISA kit, RayBiotech, Norcross, GA, USA).

### 2.4. Statistical Analysis

The statistical study was carried out using R statistical software version 4.0.4 [16] Sample size was calculating for the main outcome variable (HA), using the pwr.t2n.test() function of the pwr package for groups of different sample sizes [17]. An alpha error of 95%, a beta error of 80% were considered. The alternative hypothesis was considered “greater than”. In a pilot study, Zα and Zβ were calculated, and they were 2.57 and 1.64, respectively. Using these data, the calculated sample size was 19 study subjects.

The normality of the variables was verified with a Shapiro–Wilk test. The homoscedasticity was studied using the Levene test. None of the studied variables (VASC, VASD, EPCOJ, EVAP, HA, HAP, NO, and PON-1) met normality and homoscedasticity criteria. Because of this, a robust statistical approach and a generalized linear model were chosen for studying them.

Comparison between the studied variables (VASC, VASD, EPCOJ, EVAP, HA, HAP, NO, and PON-1), time (VISITS), and PROTOCOL (HA and PCB) were performed using bwtrim() function, included in the WRS2 package [18]. This function computes a two-way between-within subjects ANOVA on the trimmed means. At last, a general linear model (glm) was performed for study the relationship between the functional variables (VASC, VASD, EPCOJ, EVAP), time (VISITS), and protocols. For HA, HAP, NO, and PON-1 variables, for each treatment a comparison between V_1_ and V_4_ was done, and for each visit, between treatments. They were performed using the yuend() and yuen() functions for dependent and independent samples t-tests on robust location measures including effect sizes, respectively of the WRS2 package [18].

## 3. Results

Of the 58 selected dogs, 55 (27 females and 28 males) were included in the study and randomized into group A (*n* = 25) and group B (*n* = 30). Mean age was 4.69 years (dogs between 1 and 9 years of age), and mean weight was 29.6 kg (dogs between 15 and 45 kg). Three dogs were excluded from the study: one due to minor complications, one because of a major complication (implant failure), and one after developing a systemic disease during the study period (meningitis at 6 weeks).

### 3.1. Clinical Assessments

**Figure 2:**
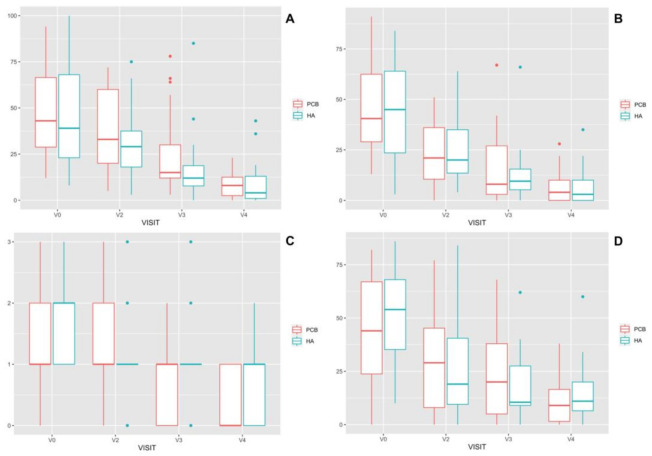
Box and whisker plots for lameness (VAS) (**A**), pain (VAS) (**B**), lameness semi-quantitative scale (**C**) evaluation by veterinarian, and pain (VAS) (**D**) evaluation by the owner; by protocol and visit. Median, range and outliers.

Both groups showed a significant decrease in lameness and pain values over time, as reported by both the owners and the veterinarians (Figure 2).

### 3.2. Biomarkers in Synovial Fluid

Total biomarker concentrations in synovial fluid prior to surgery are shown in Table 2.

At baseline (V_1_) there were no significant differences (*p* < 0.05) between the two groups (Table 2) in synovial fluid concentrations of HA, HAP, PON-1, and NO (Table 3).

After 10 weeks of treatment (V_4_), group HA showed a significant increase in HA concentration compared to baseline (V_1_) (*p* = 0.0016), while HA decreased in the PCB group over time. No significant differences were observed between groups for HA (Table 3).

After HA analyses, not all remaining samples provided enough volume to quantify all biomarkers. Therefore, they were measured according to the above-mentioned order. Similarly, in GROUP HA there was a significant decrease in PON-1 concentrations between V1 and V4 (*p* = 0.011), which did not occur in the PCB group (*p* = 0.055). No significant differences were observed between groups for PON-1. No other significant differences were found between groups or over time (Table 3).

## 4. Discussion

This study in dogs with stifle OA secondary to CCL injury shows the beneficial effect of postoperative oral HMW HA administration. More specifically, improvements in selected biomarkers, namely increased HA and decreased PON-1 concentrations in synovial fluid, were observed. The data thus support the use of such intervention as a complementary tool for managing synovitis progression in dogs after TTA surgery. Similarly, this study also examines the concentrations of several biomarkers (HA, HAP, PON-1, and NO) in the stifle of dogs with CCL injury, providing values that can serve as a reference for further studies.

The use of HA has been widely demonstrated as a joint lubricant, analgesic [10,19], and inhibitor of the production of matrix metalloproteases and other cytokine-induced inflammatory mediators [20,21,22]. In this study, HMW-HA was chosen because of its proven effectiveness in the oral treatment of patients with advanced OA [13]. However, most studies in which HA has been used have attempted to evaluate the progression of osteoarthritis or synovitis in the medium or long term, with no studies evaluating the early response. HA has traditionally been administered in dogs using the parenteral (intravenous or intra-articular) route, despite its associated disadvantages such as the need for repeated injections [23], frequent visits to the veterinarian, and possible infections associated with intra-articular injections [24]. HA oral absorption and bioavailability have previously been confirmed and this route of administration should not involve any of these complications. Balogh et al. [11] and Kimura et al. [25] reported in their studies that HA is absorbed in the small intestine when orally administered. Balogh et al. reported that ^99m^technetium-labeled HA (MW, 1 × 10^6^) is accumulated in tissues such as joints after oral administration in rats and dogs [11]. Kimura’s study demonstrated that orally administered HA is degraded to oligosaccharides by intestinal bacteria, and oligosaccharide HA is absorbed in the large intestine and subsequently distributed throughout the tissues, including the skin [25]. Therefore, oral administration should be considered a better choice for HA administration in canines.

The model chosen in this study was the evaluation of synovitis secondary to CCL injury in dogs. CCL injury is one of the leading causes of lameness in dogs. This model has been widely studied and standardized, allowing researchers to evaluate the effects of OA treatment [26]. Additionally, sampling of synovial fluid in these patients with fine needle arthrocentesis is relatively simple, has a low complication rate, and has no negative impact on the quality of the synovial fluid. Moreover, the stifle of these selected patients is large enough to allow obtaining large amounts of synovial fluid [27]. Nevertheless, it should be borne in mind that patients with CCL injury have different clinical presentations that could affect the results, such as the presence or absence of meniscal injury, varying time since the injury occurred, differing degrees of OA, and multiple types of ligament injury (lesion, partial, or complete rupture) [28,29]. However, as noted in the results, in this study the groups were homogeneous, showing no significant differences in the variables evaluated at baseline.

Tibial tuberosity advancement was chosen as the surgical technique because it has been described in detail, and its effectiveness for the treatment of CCL injury has been endorsed by numerous studies [15,29,30]. Furthermore, it is a procedure the study authors felt very familiar with, based on their extensive experience. All patients previously underwent arthrotomy to evaluate the meniscus, and in case of evidence of injury, partial meniscectomy was performed. There is some controversy regarding the need to evaluate the meniscus and its effect on the subsequent clinical evaluation [28,29], but in this case the authors performed this procedure because an untreated meniscal injury could affect measurement of the synovial biomarkers, regardless of their clinical correlation.

Clinical examination of the patient was carried out with a dual objective; on one hand, the veterinarian monitored the clinical evolution of the patient, while on the other, the owners reported their perception of such evolution. Both groups showed a significant improvement over time, with no differences found between them in any of the clinical parameters evaluated. Two conclusions may be drawn from these findings: (1) TTA is a very effective technique usually associated with excellent postoperative results, as already reported in previous papers [31,32], and (2) the surgical technique was performed correctly, achieving its purpose, which is to neutralize the caudo-cranial displacement of the tibia with respect to the femur—This was observed after the negative result of the tarsal flexion test post-surgery and at each study time point [33].

Several biomarkers were measured post-op in the study. The main one was HA concentration in synovial fluid. This is a glycosaminoglycan synthesized by chondrocytes and synovial fibroblasts [34], and it is the main component of the synovial fluid and extracellular matrix of cartilage [35]. In osteoarthritic joints, HA concentration and molecular weight decrease due to fragmentation and altered synthesis [36,37,38]. Prior studies have confirmed that orally administered HMW-HA reaches the joints as the target organ, modulating its presence as well as stimulating endogenous production [10]. Measurement of HA in synovial fluid was therefore essential for determining whether exogenous administration of HMW-HA could lead to increase HA concentrations in synovial fluid after a period of 10 weeks. Data from the study confirm that oral HA administration results in increased HA within the stifle joints of dogs undergoing TTA.

PON-1 has been recognized as an antioxidant enzyme because it hydrolyses lipid peroxides in oxidized lipoproteins. Increased synovial fluid PON1 concentrations have been reported in dogs with OA [39], while, in humans, some authors have reported increased PON1 serum concentrations [40] as OA progresses and others found decreased values [41,42]. In the present study, PON-1 synovial fluid concentrations decreased over time in group HA and there were no significant differences between groups. This finding suggests an anti-inflammatory effect of oral HMW-HA in acute stages of OA, similarly to what has been described previously [19,20,21]. Although this finding can be supported by the results of the present study and those of other authors that report an anti-inflammatory action of exogenous HA, it should be approached with caution, and confirmed by further studies.

HAP, a moderate acute-phase protein, was chosen because it acts as an inflammatory mediator, and has shown prognostic and diagnostic value for early identification of inflammation and for the prevention of progression of the osteoarthritic process [43,44]. Nevertheless, the authors found no variations in its synovial concentration over time or between treatments.

NO is the principal and most widely studied gaseous inflammatory mediator [45,46] with activity in osteoarthritic processes [47,48]. NO was chosen for its role in the regulation of vascular tone and as an endogenous vasodilator [49], as well as for its critical role in the production and reduction of nociception and pain [50]. However, in this study, the authors found no variations in its synovial concentration over time or between treatments, although the values obtained in the study are comparable to those of other authors [51] who have found substantially higher values than those for healthy animals.

The main limitation of the study was the variability in degree of OA among study subjects at time of surgery (V_1_). In addition, another limitation is the duration of follow-up, since OA is a chronic disease. Therefore, the authors feel that it would be of interest to evaluate the effect of HMW-HA after a longer period of oral administration.

## 5. Conclusions

In conclusion, the results demonstrated that postoperative oral administration of HMW-HA in canine patients with OA secondary to CCL injury leads to improvements in OA biomarkers measured in synovial fluid, specifically in HA and PON-1 concentrations. Additionally, this paper also reports the concentrations of various biomarkers (HA, HAP, PON-1, and NO) in the stifle of dogs with CCL injury, providing reference values for subsequent studies.

## Figures and Tables

**Table 1:** Summary of experimental protocol.

Visit	V_0_	V_1_	V_2_	V_3_	V_4_
Diagnostic consultation (specialty consultation, radiology, and blood tests)	X				
Signing of informed consent (study information and document signing)	X				
Functional assessment (assessment by owner and veterinarian)	X		X	X	X
TTA surgery (surgical treatment, postoperative radiology, and arthrocentesis)		X			
Synovial fluid aspiration		X			X

V_0_: Consultation at which the diagnosis was confirmed, V_1_: Day of surgery, baseline for biomarker concentrations V_2_: Check-up 2 weeks after surgery, V_3_: Check-up 4 weeks after surgery, V_4:_ Check-up 10 weeks after surgery, X: visit at which each step of the experimental protocol was performed.

**Table 2:** Synovial fluid biomarker concentrations at baseline (V_1_). Values represent synovial concentration of biomarkers in the stifle of dogs with cranial cruciate ligament lesion.

Biomarker (Units)	Mean ± SD
HA (µg/mL)	1614.62 ± 393.60
HAP (g/L)	0.22 ± 0.36
NO (µmol/L)	9.38 ± 7.57
PON-1 (IU/mL)	0.86 ± 0.61

HA: Hyaluronic acid; HAP: haptoglobin; NO: nitric oxide; PON-1: paraoxonase-1.

**Table 3:** Synovial fluid biomarker concentrations in different groups.

Biomarker		Group PCB		Group HA	
		*n*	Median (Min–Max)	*n*	Median (Min–Max)
HA (μg/mL)	V_1_	23	1810 (952–2090)	30	1670 (301–2230) ^a^
	V_4_	21	1650 (1240–2030)	19	1780 (993–2730) ^a^
PON-1 (IU/mL)	V_1_	19	0.91 (0.14–3.10)	23	0.67 (0.14–1.64) ^b^
	V_4_	16	0.14 (0.12–1.11)	19	0.14 (0.13–1.87) ^b^
NO (µmol/L)	V_1_	17	8.47 (3.08–150)	19	8.30 (1.96–81.80)
	V_4_	17	6.26 (1.80–78.70)	13	5.38 (2.61–97.50)
HAP (g/L)	V_1_	22	0.15 (0.01–1.29)	22	0.03 (0.01–1.66)
	V_4_	21	0.02 (0.01–1.53)	21	0.02 (0.01–1.50)

HA: Hyaluronic acid; HAP: haptoglobin; NO: nitric oxide; PON-1: paraoxonase-1. V_1_: Day of surgery, V_4_: Check-up 10 weeks after surgery. n: sample size for every variable at every group and visit. Same super index letter means significant differences with *p* < 0.05. ^a^ HA concentration differences in GROUP HA between V_1_ and V_4_. ^b^ PON-1 concentration differences in GROUP HA between V_1_ and V_4_.

## Data Availability

The datasets used and/or analyzed during the current study are available from the corresponding author on reasonable request.
